# Assessing the Risks of Topically Applied dsRNA-Based Products to Non-target Arthropods

**DOI:** 10.3389/fpls.2020.00679

**Published:** 2020-06-04

**Authors:** Jörg Romeis, Franco Widmer

**Affiliations:** ^1^Research Division Agroecology and Environment, Agroscope, Zurich, Switzerland; ^2^Competence Division Method Development and Analytics, Agroscope, Zurich, Switzerland

**Keywords:** ecosystem services, environmental risk assessment, hazard, exposure, pathways to harm, problem formulation, species selection, tiered risk assessment

## Abstract

RNA interference (RNAi) is a powerful technology that offers new opportunities for pest control through silencing of genes that are essential for the survival of arthropod pests. The approach relies on sequence-specificity of applied double-stranded (ds) RNA that can be designed to have a very narrow spectrum of both the target gene product (RNA) as well as the target organism, and thus allowing highly targeted pest control. Successful RNAi has been reported from a number of arthropod species belonging to various orders. Pest control may be achieved by applying dsRNA as foliar sprays. One of the main concerns related to the use of dsRNA is adverse environmental effects particularly on valued non-target species. Arthropods form an important part of the biodiversity in agricultural landscapes and contribute important ecosystem services. Consequently, environmental risk assessment (ERA) for potential impacts that plant protection products may have on valued non-target arthropods is legally required prior to their placement on the market. We describe how problem formulation can be used to set the context and to develop plausible pathways on how the application of dsRNA-based products could harm valued non-target arthropod species, such as those contributing to biological pest control. The current knowledge regarding the exposure to and the hazard posed by dsRNA in spray products for non-target arthropods is reviewed and suggestions are provided on how to select the most suitable test species and to conduct laboratory-based toxicity studies that provide robust, reliable and interpretable results to support the ERA.

## Introduction

RNA interference (RNAi) is a mechanism of gene silencing present in most eukaryote organism to regulate gene expression (Hannon, 2002). The silencing effect can be triggered by double-stranded RNA (dsRNA), is RNA sequence-specific, and makes use of the core RNAi machinery to degrade complementary RNA molecules. RNAi thus provides a tool that can be designed to affect and control insect pests in a highly specific manner by targeting genes that are essential for the survival of the species (Xue et al., 2012; Burand and Hunter, 2013; Zhang et al., 2017; Liu et al., 2020). In an agricultural context the technology may also be deployed to increase the sensitivity of pests or vectors to chemical insecticides (e.g., Killiny et al., 2014; Bona et al., 2016) or to protect beneficial species from viral diseases (Vogel et al., 2019).

For application as a pest control tool, the active dsRNA molecule has to enter and affect the target pest. This can be achieved by two main ways of application. First, dsRNA can be produced *in planta*, which requires genetic engineering (GE) of the plant. The first product of that kind has recently been approved by US regulators in June 2017^1^. This particular GE maize event (MON87411) produces a dsRNA targeting the Snf7 protein in the Western Corn Rootworm, *Diabrotica virgifera virgifera* (Coleoptera: Chrysomelidae), which is crucial for the transport of transmembrane proteins. Suppression of the *Snf7* gene leads to increased larval mortality and consequently to reduced root damage (Bolognesi et al., 2012). The RNAi trait is combined with the Cry3Bb1 protein for improved target pest control and resistance management (Levine et al., 2015; Head et al., 2017). Second, the dsRNA molecules can be applied externally, for example in irrigation water or through trunk injections (Hunter et al., 2012; Li et al., 2015a; Niu et al., 2018; Kunte et al., 2020), in food-baits (Zhou et al., 2008; Zhang et al., 2010), by using delivery systems such as micro-organisms, viruses, nanocarriers (Kunte et al., 2020; Vogel et al., 2019), or topically as spray applications (San Miguel and Scott, 2016).

Two major challenges have been identified for implementing the RNAi-based technology in pest control. First, the target organisms have to ingest intact and biologically active dsRNA molecules in order to trigger an RNAi response. While RNAi has been observed in a number of insect species belonging to various orders, the effectiveness of dietary RNAi (derived from ingested dsRNA) is less clear (Baum and Roberts, 2014). Second, there is evidence that resistance is not developed against a specific dsRNA molecule but to components in the dsRNA uptake machinery in the intestinal tract or in the dsRNA processing machinery. For example, Khajuria et al. (2018) demonstrated for *D. v. virgifera*, that resistance to dsRNA targeting *Snf7*, was due to the fact that cellular uptake was prevented.

Despite those challenges, effective dsRNA-based spray products that cause specific toxic effects on selected arthropod pest species are expected within the next few years (Hogervorst et al., 2018; Taning et al., 2020) and our perspective will focus on this method of application.

## Environmental Risk Assessment

As pesticides, dsRNA-based sprays are regulated stressors that have to pass an environmental risk assessment (ERA) before being commercially released to ensure that their use causes no unacceptable harm to the environment. Given the novel mode of action, the regulatory and data requirements are discussed internationally (Auer and Frederick, 2009; US EPA, 2014; Roberts et al., 2015).

Early in the ERA, in a step called “Problem Formulation,” the protection goals set by environmental policy need to be identified, and operational protection goals and plausible pathways on how the stressor of concern could harm those protection goals (i.e., pathways to harm) are defined (Raybould, 2006; Gray, 2012; Craig et al., 2017; Raybould et al., 2019). Based on these “Pathways to Harm,” testable risk hypotheses can be derived, existing relevant information is collected and required data are identified. The aim of this process is to ensure that any decision taken is made in a traceable and transparent manner. While experience has been gained with applying problem formulation to the ERA of GE plants, the concept is equally applicable to other stressors, including dsRNA-based pesticides (Devos et al., 2019; Raybould and Burns, 2020).

For plant protection products such as dsRNA-based sprays, “biodiversity” is an important environmental protection goal, which is found in policies of most jurisdictions. However, this term is very general and thus specific (operational) protection goals need to be defined that can then be addressed in the scientific risk assessment. Such operational protection goals delineate the components of the environment that are valued and should be protected, including details on the location, the exact time period, and the maximum tolerable impact (Nienstedt et al., 2012; Sanvido et al., 2012; Devos et al., 2015). In this respect, it has been proposed to categorize biodiversity in categories of valued ecosystem services (“ecosystem service concept”) as defined for example in the Millennium Ecosystem assessment (Millennium Ecosystem Assessment [MEA], 2005; Gilioli et al., 2014; Devos et al., 2015; European Food Safety Authority Scientific Committee, 2016; Maltby et al., 2017a, b). In the case of arthropods this includes regulating services (e.g., biological pest control, pollination), cultural services (e.g., protected species), and supporting services (e.g., arthropods that contribute to nutrient cycling).

**FIGURE 1:**
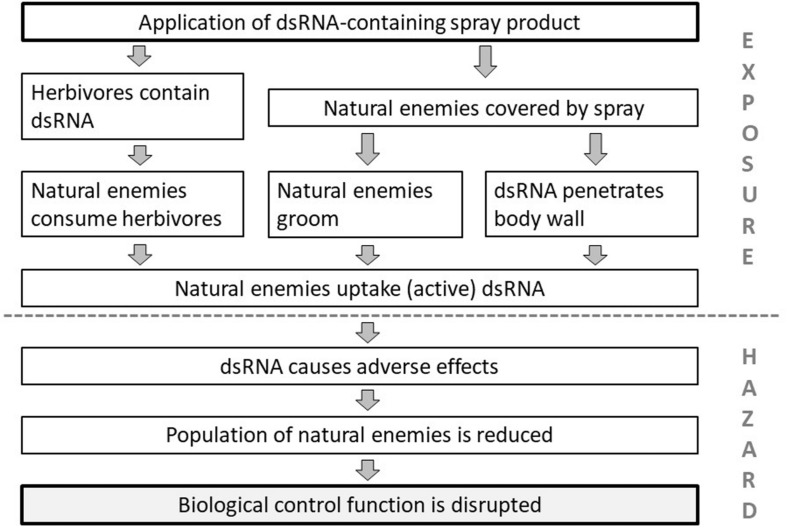
Plausible pathways to harm. Steps on how the application of a dsRNA-based spray insecticide could cause harm to the protection goal of “biological pest control” by affecting arthropod natural enemies (predators and parasitoids).

Once the components of the environment to be protected are identified, plausible pathways to harm can be constructed. In Figure 1 such pathways to harm are defined for the protection goal “biological pest control” that is provided by predators and parasitoids, which may be affected by the application of a dsRNA-based spray. For a spray product to cause harm to the protection goal, a line of events or steps has to occur. If one can conclude with high certainty that one or more of the steps are unlikely to happen, the pathway is interrupted, which allows to conclude that the risk to biological control is negligible (Raybould et al., 2019). Thus the different steps can be tested or assessed in the ERA to characterize the risk. In principle the steps either relate to exposure, the likelihood that non-target species actually ingest sufficient amounts of biological active dsRNA, or hazard, which relates to the sensitivity of the non-target species to dietary RNAi. These two aspects of the risk equation will be discussed in the following sections.

## Exposure of Non-Target Arthropods to dsRNA in Spray Products

Non-target arthropod species could directly be exposed to dsRNA in spray products when consuming treated plant material in the field or outside the field in case of spray-drift, through contact with soil and water or topical application and indirect when feeding on arthropods that have been exposed. While the plant cuticle and also the cell walls limit the uptake of spray-applied dsRNA into the plants, there is some evidence for uptake and transport in the vascular system of bioactive dsRNA (Koch et al., 2016), which can be further enhanced by high pressure spraying (Dalakouras et al., 2016) or particular carriers (Mitter et al., 2017).

In general, stability of naked dsRNA in the environment is very low. Degradation of dsRNA within 2 days has been reported for soil and aquatic environments (Dubelman et al., 2014; Fischer et al., 2016, 2017; Bachman et al., 2020) although partial adsorption to soil particles will also play a role (Parker et al., 2019). Degradation appears neither to be affected by dose (Dubelman et al., 2014) nor by length or structure of the dsRNA molecule (Fischer et al., 2016). There is some indication that degradation of dsRNA molecules is reduced on plant surfaces (Tenllado et al., 2004; San Miguel and Scott, 2016). The persistence of dsRNA in formulated spray products is difficult to predict since the active ingredient is likely to be stabilized to prevent abiotic and biotic degradation. For example, Mitter et al. (2017) recently demonstrated that pathogen-specific dsRNA targeting plant viruses could be detected for more than 30 days after application when loaded on layered double hydroxide clay nanosheets. Thus, the formulation in which the molecule is applied has to be considered in the exposure assessment (Bachman et al., 2020).

The routes and duration of non-target organism exposure to dsRNA in sprayed products will depend on a number of factors, including: (1) application rate of the active ingredient, (2) application timing, (3) application method, (4) number of applications, (5) off-site movement of applied dsRNA, and (6) stability and persistence of exogenously applied dsRNA following application (US EPA, 2014).

For predators and parasitoids we have identified three main routes of exposure (Figure 1). The first, and the most likely route is indirect, through their prey or hosts. Herbivores can be covered by the spray or ingest the dsRNA when feeding on the treated plants. It remains to be confirmed, however, that dsRNA ingested by a herbivore is still biologically active when passed on to the next trophic level. To our knowledge, cross-species transfer of biologically active dsRNA has only been reported in one study, i.e., between honey bees (*Apis mellifera*, Hymenoptera: Apidae) and parasitic mites, *Varroa destructor* (Acari: Varroidae) (Garbian et al., 2012). The second potential route of exposure of natural enemies is through the insects’ integument. There is some evidence that dsRNA applied topically can penetrate the insect’s body wall, i.e., via the inter-segmental membranes, and cause an RNAi response. The first case of this nature was reported for *Aedes aegypti* (Diptera: Culicidae) by Pridgeon et al. (2008). Penetration has also been demonstrated for larvae of *Ostrinia furnacalis* (Lepidoptera: Crambidae) using fluorescent dsRNA albeit at very high concentrations of 0.5 μl of 0.5 μg/μl fluorescent labeled dsRNA per larva (Wang et al., 2011). However, it is difficult in such topical application studies to rule out that the dsRNA molecules entered the body through the spiracles rather than through the integument. However, there is evidence that the penetration efficiency can be enhanced by altering the formulation in which the dsRNA is applied. For example, in the case of the soybean aphid *Aphis glycines* (Hemiptera: Aphididae) penetration efficiency was significantly enhanced using a nanocarrier in combination with an amphiphilic periphery detergent to increase the attachment of the droplets to the insect cuticula (Zheng et al., 2019). In a recent study, Niu et al. (2019) observed the uptake of dsRNA topically applied to *Acyrthosiphon pisum* (Hemiptera: Aphididae) within 12 min. As a third route of exposure, insects might also ingest the molecule during grooming after they have been covered by dsRNA after a spray application. While some predators also feed on green plant tissue when prey is scarce (Lundgren, 2009) we regard this route of exposure as negligible.

Dietary uptake of dsRNA, does not necessarily mean that the molecule is still biologically active. Extraoral digestion is know from many predatory arthropods including spiders, lacewing larvae and predatory bugs (Cohen, 1998; Zhu et al., 2016; Walter et al., 2017). According to Cohen (1995) at least 79% of predaceous land-dwelling arthropods use extra-oral digestion. For example, it has been demonstrated for the plant bug *Lygus lineolaris* (Hemiptera: Miridae) that dsRNA molecules are completely digested to monomers by endonucleases in the saliva prior to ingestion (Allen and Walker, 2012).

## Hazard Posed by dsRNA

In principle, ingested dsRNA can pose a hazard to a non-target arthropod in two ways, i.e., sequence-specific and sequence-unspecific. Mechanisms that have been suggested as a cause of sequence-unspecific effects of ingested dsRNA are first, the induction of a general immune response since RNAi is a component of the innate antiviral immunity response and second, a saturation of the RNAi machinery, i.e., the dsRNA processing enzymes (Dillin, 2003; Christiaens et al., 2018a). While saturation of the RNAi machinery has been observed in animals (mice and cell cultures) at high doses (US EPA, 2014), it has not yet been reported in arthropods (Miller et al., 2012; Christiaens et al., 2018a). DsRNA-triggered general immune responses, e.g., the upregulation of dsRNAase, have been observed in honey bees (*Apis mellifera*, Hymenoptera: Apidae) (Flenniken and Andino, 2013; Brutscher et al., 2017), bumble bees (*Bombus terrestris*, Hymenoptera: Apidae) (Piot et al., 2015), and the silkworm (Liu et al., 2013). There is evidence from feeding studies that high doses of dsRNA can boost a sequence-unspecific response in ladybird beetles (Coleoptera: Coccinellidae) (Haller et al., 2019). But comparable doses (of the same construct) did not cause such effects in other arthropod species studied (Pan et al., 2016; Vélez et al., 2016). Sequence-unspecific effects have also been observed for dsGFP in honey bees, *A. mellifera*, in feeding and injection studies (Jarosch and Moritz, 2012; Nunes et al., 2013). In summary, while there is no evidence that dsRNA can cause a saturation of the RNAi machinery in arthropods, high doses of dsRNA may affect the fitness of non-target arthropod species in a sequence-unspecific way through a stimulation of the immune system. Consequently, from an ERA perspective, non- and off-target effects of the dsRNA that are sequence specific are of much more concern and will be the focus of the following description.

After ingestion of dsRNA molecules, a successful RNAi response depends on a variety of factors that will be discussed below, including: stability of dsRNA in the gut (affected by gut pH and nucleases), dsRNA length and concentration, target gene, arthropod species and the life-stage exposed (Katoch et al., 2013; Scott et al., 2013; Davis-Vogel et al., 2018; Cooper et al., 2019; Kunte et al., 2020).

Once an insect has ingested dsRNA and the molecule has been taken up by the cells, the endonuclease Dicer cuts the molecule into short interfering RNAs (siRNA) of a length of 20–25 bp that are integrated into the RNA-induced silencing complex (RISC) (Hannon, 2002). Subsequently RISC facilitates the targeting and the endonucleolytic attack on mRNAs with sequence identity to the dsRNA (Hannon, 2002). The pre-requisite for a successful RNAi response is thus sequence identity between at least some of the siRNAs derived from the dsRNA and the target mRNA of the insect pest (Scott et al., 2013). Consequently, length of the dsRNA affects the effectiveness of the RNAi response, as longer molecules yield larger populations of overlapping siRNA molecules ranging in size and sequence (Baum et al., 2007; Bolognesi et al., 2012; Miller et al., 2012; Li et al., 2015b; Nandety et al., 2015). An injection study with *Tribolium castaneum* (Coleoptera: Tenebrionidae) suggests that the size of the dsRNA molecule also affects the duration of the RNAi response, event though the mechanism involved remains unclear (Miller et al., 2012). There is evidence that contiguous sequence matches of ≥21 nt of the dsRNA to the target gene are necessary for dsRNA to be biologically active in insects (Bachman et al., 2013, 2016; Roberts et al., 2015) and it has been reported that even a single 21 nt sequence match can induce effects (Bolognesi et al., 2012). It has to be noted, however, that RNAi has been demonstrated to occur at sequence length as short as 15 bp (Powell et al., 2017). Still uncertain is the extent of sequence mismatch that has to be present in order to prevent dsRNA-derived siRNAs. Because siRNA molecules can inhibit translation of transcripts even when mismatches occur, the threshold for concern about non-target effects could be less than 100% sequence identity (Scott et al., 2013). For providing the evidence that any observed effect is due to specific gene silencing, it is necessary to support the feeding assays by determination of transcript levels with RT-qPCR. This, however, poses the challenge of identifying suitable reference or housekeeping genes to calculate relative transcript levels. Furthermore, the effect of RNAi on the protein may not be well correlated to the level of transcript suppression (Scott et al., 2013).

While functional RNAi has been reported from a number of insect species belonging to various orders, the impact of dietary RNAi is more limited (Baum and Roberts, 2014). While many insects have been found to be susceptible to dietary RNAi (Belles, 2010), large differences in sensitivity have been reported across taxa (Whangbo and Hunter, 2008; Terenius et al., 2011; Cooper et al., 2019). For example, feeding studies where solutions containing dsRNA were provided demonstrated that many Coleoptera show a LC_50_ at dsRNA concentrations from 1 to −10 ppb, while effects are seen in Diptera at 10–500 ppm, and in Lepidoptera/Hemiptera at > 1000 ppm (Baum and Roberts, 2014). It has to be noted, however, that sensitivity to dietary RNAi can vary significantly among even closely related species as has been demonstrated for sweetpotato weevils, *Cylas* spp. (Coleoptera: Brentidae) (Christiaens et al., 2016; Prentice et al., 2017). It can even vary between strains/populations of a particular species as has for example been reported for *Locusta migratoria* (Orthoptera: Acrididae) (Sugahara et al., 2017) and *T. castaneum* (Kitzmann et al., 2013; Spit et al., 2017).

Degradation of the dsRNA after ingestion or uptake is a major factor affecting the exposure of non-target species to bioactive dsRNA molecules and thus the effectivity of RNAi (Wang et al., 2016). Gut pH is important as it affects the stability of the ingested dsRNA molecules. Since RNA is most stable at pH of 4.0–5.0, the slightly acidic midguts of Coleoptera and Hemiptera (pH around 5) support dsRNA stability. In contrast, stability is low in the alkaline guts of Orthoptera, Diptera and Hymenoptera and in particular in the highly alkaline guts of Lepidoptera (pH > 8.0) (Cooper et al., 2019). In addition, dsRNA can be degraded by nucleases in the insect guts as has for example been reported for *Bombyx mori* (Lepidoptera: Bombycidae) (Arimatsu et al., 2007; Liu et al., 2012, 2013) and the desert locust, *Schistocerca gregaria* (Orthoptera: Acrididae) (Wynant et al., 2014). Degradation of dsRNA in the gut also explains the relatively low sensitivity of *Cylas puncticollis* to dietary RNAi when compared to the closely related *C. brunneus* (both Coleoptera: Brentidae) (Christiaens et al., 2016; Prentice et al., 2017). After uptake, dsRNA can be degraded by nucleases in the haemolymph (Wang et al., 2016) as has for example been reported for *Manduca sexta* (Lepidoptera: Sphingidae) (Garbutt et al., 2013) and *A. pisum* (Christiaens et al., 2014).

To enhance the stability of the ingested dsRNA, to prevent degradation by nucleases and to enhance cellular uptake, various carriers have successfully been deployed (Yu et al., 2013; Christiaens et al., 2018b; Kunte et al., 2020; Vogel et al., 2019). This includes lipid-based encapsulations (Whyard et al., 2009; Taning et al., 2016; Lin et al., 2017), cell-penetrating peptides (Gillet et al., 2017), polymers (Zhang et al., 2010; Christiaens et al., 2018a), and other nanoparticles (He et al., 2013; Das et al., 2015). In addition the RNAi response can be enhanced by co-delivery of nuclease-specific dsRNA (Spit et al., 2017; Cooper et al., 2019). Thus, the formulation in which the dsRNA is provided also has to be considered when judging the hazardous potential of the molecule to non-target species.

## Selection of Test Species for Non-Target Studies

Since not all valued non-target arthropods present in the receiving environment that are potentially exposed to the dsRNA-based product can be tested, surrogate (test) species need to be selected for toxicity studies to support the non-target risk assessment. The following description focuses on the selection of test species to detect sequence-specific effects caused by the particular dsRNA molecule under consideration.

Non-target testing of chemical pesticides has a long history in Europe. At the initial stage, only 2 species are tested under worst-case exposure conditions, i.e., the predatory mite *Typhlodromus pyri* (Acari: Phytoseiidae) and the parasitic wasp *Aphidius rhopalosiphi* (Hymenoptera: Braconidae) (Candolfi et al., 2001). The two species were selected as indicators since sensitivity analyses revealed that they are the most sensitive species to most classes of pesticides (Candolfi et al., 1999; Vogt, 2000). Consequently, by testing those species predictions of effects on other non-target arthropods can be made with high confidence (Candolfi et al., 1999). Only if adverse effects above a certain threshold are detected for those species and unacceptable risk can thus not be excluded additional tests with other beneficial species are indicated. These include *Orius laevigatus* (Hemiptera: Anthocoridae), *Chrysoperla carnea* (Neuroptera: Chrysopidae), *Coccinella septempunctata* (Coleoptera: Coccinellidae), and *Aleochara bilineata* (Coleoptera: Staphilinidae). These species were selected because they are commercially available, amenable to testing in the laboratory, reliable test protocols exist, they provide sufficient phylogenetic and functional diversity, and common in agricultural fields (Barrett et al., 1994; Candolfi et al., 2001). In addition to testing predators and parasitoids, most regulatory jurisdictions (e.g., European Commission [EC], 2002), require testing of honey bees (*A. mellifera*) and soil organisms [*Folsomia candida* (Collembola: Isotomidae) or *Hypoaspis aculeifer* (Acari: Gamasidae)], if exposure of the latter is anticipated.

This common set of surrogate test species, however, is not suitable to assess non-target effects caused by dsRNA-based spray products because the initial two indicator species were selected for their sensitivity to chemical pesticides but are unlikely to be the most sensitive species for the majority of dsRNA molecules. Consequently it would be more suitable to apply the approach for non-target risk assessment as is conducted for GE plants expressing insecticidal proteins, such as Bt crops expressing Cry or VIP proteins from *Bacillus thuringiensis*. The ERA for GE plants is conducted case-by-case and consequently the most appropriate non-target species can be selected for each plant/trait combination. It has been proposed to base the selection of test species for laboratory studies on three main criteria (Romeis et al., 2013):

*(i) Sensitivity*: species should be the most likely to be sensitive to the stressor under consideration based on the known spectrum of activity, its mode of action, and the phylogenetic relatedness of the test and target species.

*(ii) Relevance*: species should be representative of valued taxa or functional groups that are most likely to be exposed to the stressor in the field. Organisms that contribute to important ecosystem service and are considered relevant have been identified for a number of field crops (e.g., Meissle et al., 2012; Romeis et al., 2014; Riedel et al., 2016; Li et al., 2017).

*(iii) Availability and reliability*: suitable life-stages of the test species must be obtainable in sufficient quantity and quality, and validated test protocols must be available that allow consistent detection of adverse effects on ecologically relevant parameters. Lists of above-ground, below-ground, and aquatic species that are available and amenable for testing have been published (e.g., Candolfi et al., 2000; Römbke et al., 2010; Romeis et al., 2013; Carstens et al., 2012; Li et al., 2017).

The above listed criteria are also key elements of other test species selection approaches that have for example been published by Todd et al. (2008) and Hilbeck et al. (2014).

While the criteria (ii) and (iii) are relative generic or crop-specific, criteria (i) needs to be addressed specifically for each stressor under consideration. To increase the robustness and reliability of the non-target risk assessment the species most likely to be sensitive (= affected) to a particular dsRNA should be selected. This includes considerations of the gene or gene family that is targeted and the knowledge about the sensitivity of certain taxa to dietary RNAi in general. The phylogenetic relationship of the non-target organisms to the target pest should also be considered, as there is evidence that, in general, species closely related to the target organism are more likely to be susceptible to the dsRNA than distantly related species (Whyard et al., 2009; Bachman et al., 2013, 2016; US EPA, 2014; Roberts et al., 2015).

Since the RNAi response is sequence specific, bioinformatics can help predicting the species most likely affected that could then be used in feeding studies (Bachman et al., 2013, 2016). However, it has to be recognized that the presence of sequence homologies between the dsRNA molecule and the genome of the non-target species does not necessarily indicate sensitivity of an organisms. For example, the springtail *Sinella curviseta* (Collembola: Entomobryidae) shares a total of six 21 nt long matches with the dsRNA targeting the *vATPase A* in *D. v. virgifera*. However, the organism was not adversely affected in laboratory feeding studies (Pan et al., 2016). In cases where for some reason (species that are rare, protected or difficult to rear), bioinformatics may, however, be the only way to “test” the species (Bachman et al., 2016). Bioinformatics could also help predicting off-target effects. However, currently we lack genomic data for most non-target species. It would be useful to have more genome data available for model non-target species that actually play a role in agricultural production systems to effectively apply bioinformatics to the NTO risk assessment (Casacuberta et al., 2015; Fletcher et al., 2020).

## Design and Implementation of Non-Target Laboratory Toxicity Studies

The established test protocols published by the West Palaearctic Regional Section of the International Society for Biological and Integrated Control (IOBC/WPRS; Candolfi et al., 2000) or by the European and Mediterranean Plant Protection Organization (EPPO)^2^ for early-tier laboratory toxicity studies for chemical insecticides are based on contact toxicity. Those test protocols thus do not allow assessing the non-target effects of dsRNA for which oral uptake is the most important route of exposure. The lack of standardized test protocols addressing the oral route of exposure and to detect effects resulting from novel modes of action has recently been pointed out by the Panel on Plant Protection Products and their Residues of European Food Safety Authority (2015) even though RNAi was not specifically mentioned.

However, experience is available with gut-active insecticidal proteins such as the Cry and VIP proteins from *B. thuringiensis*. Guidance exists on how to design and perform laboratory feeding studies with such proteins to provide high quality, reliable and robust data (Romeis et al., 2011; De Schrijver et al., 2016). When designing a non-target laboratory study the following main criteria should be considered (Romeis et al., 2011): (i) Test substance characterization and formulation; (ii) Method of delivery; (iii) Concentration/dose; (iv) Measurement endpoints; (v) Test duration; (vi) Control treatments; (vii) Statistical considerations.

Since the formulation in which the dsRNA is provided has a strong effect on the dsRNA uptake and the strength of the RNAi response in arthropods (as discussed above) care should be taken that the test substance is provided in a realistic formulation.

It is generally considered that toxicity of insecticidal compounds such as chemical insecticides and Cry proteins from Bt increases with increasing concentration in which they are delivered. Thus safety is added to the non-target studies by testing unrealistically high concentrations of the stressor of concern to provide a margin of safety and to account for possible intra- and interspecific variability from the use of a surrogate test species. Definition of the concentrations to be tested poses some challenges for different reasons. First, the length of the dsRNA affects the effectiveness to trigger an RNAi response (Bolognesi et al., 2012; Miller et al., 2012), thus the margins of safety may vary between constructs. Second, there is evidence that there is no clear dose-relationship but that RNAi is triggered from a specific threshold dose onward and might be maximal at an optimal dose (Turner et al., 2006; Niu et al., 2019). Third, high doses may cause sequence-unspecific effects as discussed above.

The endpoints to be recorded (lethal and sublethal) need to be selected based on the organism under investigation (and the reliability of the test system) and the gene that is targeted. While lethality is an obvious endpoint to be chosen, the consideration of sublethal endpoints such as growth or development time is recommended (Roberts et al., 2020). First, they may hint to unexpected off-target effects, second, they may cover for the fact that dsRNA is generally slow acting (Baum and Roberts, 2014) and that the process is typically not reaching 100% gene suppression (e.g., Bolognesi et al., 2012; Rangasamy and Siegfried, 2012), and third, they might address the fact that RNAi effects can be transgenerational, i.e., also affecting subsequent generations (Abdellatef et al., 2015). Sublethal endpoints are typically also recorded in the testing of chemical pesticides (e.g., Candolfi et al., 2000) and Bt proteins (De Schrijver et al., 2016; Roberts et al., 2020) even though mortality is the primary endpoint and often the results from testing sublethal endpoints are not reported in regulatory summaries. In any case, it is important to set decision-making criteria for every endpoint that is recorded. The duration of the study needs to be selected so that the measurement endpoints show a response should the test substance have an effect. Given the slow RNAi response, test probably need to be extended in duration compared to Bt Cry proteins (e.g., Bachman et al., 2013, 2016).

A key element of every laboratory study is the inclusion of a negative control treatment that allows to separate effects caused by the test system (e.g., the fitness of the test organisms, the suitability of the diet) from those caused by the test substance. Ideally, the negative control consists of a dsRNA molecule that targets a heterologous sequence absent from the insect’s genome and that does thus not lead to specific gene silencing in the test species. This would control for any impact caused by a trigger of the RNAi cascade (sequence unspecific effects). Typical examples that have been used for this purpose include dsRNA targeting the green fluorescent protein (GFP) and β-glucuronidase (GUS). However, there is some evidence, that dsGFP causes adverse effects in arthropods when applied orally at very high doses (Nunes et al., 2013; Haller et al., 2019) or when injected (Jarosch and Moritz, 2012).

Positive controls, i.e., the addition of dsRNA molecules that are designed to silence a gene in the test insects can further help to interpret the study results as they provide evidence that the test system can detect a response and that the test species is sensitive to dietary RNAi. Positive controls have for example been deployed by Haller et al. (2019) when testing the effect of dsRNA targeting the *vATPase-A* of *D. v. virgifera* in two non-target ladybird beetles (Coleoptera: Coccinellidae). The data confirmed that two species of ladybirds are sensitive to dietary RNAi but that the non-target dsRNA molecule only had a weak effect. Another study using the same test substance in honey bees did not detect any effects in the positive control treatment raising doubts about the sensitivity of honey bees to dietary RNAi in general (Vélez et al., 2016).

## Conclusion

In order to assess whether dsRNA-based pesticide sprays adversely affect valued non-target species in the agroecosystem, three questions need to be addressed: (1) Are the non-target arthropods exposed to biologically active dsRNA? (2) Do the non-target arthropods possess the RNAi machinery for dsRNA to trigger a response? and (3) are there sufficient sequence matches between the dsRNA molecule under consideration and the genome of the non-target arthropods to cause a sequence-specific effect.

While it is possible to make some generalizations regarding the level of exposure, potential uptake of dsRNA and the sensitivity to dietary RNAi for common non-target species in field crops, some open questions remain. For example it is still unclear to what extent the bioactive dsRNA molecule is transferred through the arthropod foodweb and whether penetration through the arthropod body wall is a relevant route of exposure for non-target species. Furthermore, it would be useful to evaluate whether the risk for certain arthropod taxa can be considered negligible because they digest dsRNA prior to ingestion and are thus unlikely to be exposed.

Concerning the hazard posed by dsRNA, it would be important to evaluate whether there are species or taxa that can be considered safe because they are insensitive to dietary RNAi in general (e.g., because they lack the dsRNA uptake mechanism). Also, uncertainty still exists regarding the sequence mismatches (and number thereof) between the targeted mRNA and the dsRNA that still allows for an RNAi response. There is evidence that genome information can help assess non-target effects. However, bioinformatics information is still lacking for most valued non-target arthropods. This information would help assist to predict non-target effects and select the most suitable (i.e., potentially sensitive) species to conduct feeding studies in the laboratory. Related to this, the power of bioinformatics for predicting non-target effects still needs to be further investigated before this information can be used to draw a conclusion about safety.

Consequently, it is essential to conduct feeding studies to assess whether the ingestion of dsRNA molecules poses a hazard to relevant non-target species. However, when planning the studies to be conducted in the laboratory with dsRNA-based pesticides, it would be necessary to add flexibility to the non-target risk assessment framework used for chemical pesticides to allow a case-by-case assessment as is done for GE plants. A challenge remains the selection of the most appropriate negative and positive control treatments to ensure a robust interpretation of the study results and to minimize false negative and false positive results.

The main concern, however, is the fact that the carrier to which the dsRNA is bound or the formulation in which it is applied will be of ample importance as it not only affects the level at which non-target arthropods will be exposed, i.e., the stability and distribution of the active compound in the environment and in the insect gut and body, but also the extent of the RNAi response.

While there is a lot to profit from the experience with chemical pesticides and GE plants producing insecticidal proteins, insecticidal sprays based on dsRNA still pose some specific challenges to the non-target risk assessment.

## Author Contributions

JR and FW wrote and approved the manuscript.

## Disclaimer

The opinions expressed and arguments employed in this paper are the sole responsibility of the authors and do not necessarily reflect those of the OECD or of the governments of its Member countries.

## Conflict of Interest

The authors declare that the research was conducted in the absence of any commercial or financial relationships that could be construed as a potential conflict of interest.

## Footnotes

1 https://www.epa.gov/pesticide-registration/epa-registers-innovative-tool-control-corn-rootworm

2 https://pp1.eppo.int/standards/side_effects
